# No seasonal curtailment of the Eurasian Skylark's (*Alauda arvensis*) breeding season in German heterogeneous farmland

**DOI:** 10.1002/ece3.9267

**Published:** 2022-09-20

**Authors:** Manuel Püttmanns, Franziska Lehmann, Fabian Willert, Jasmin Heinz, Antje Kieburg, Tim Filla, Niko Balkenhol, Matthias Waltert, Eckhard Gottschalk

**Affiliations:** ^1^ Department of Conservation Biology Johann‐Friedrich‐Blumenbach Institute of Zoology and Anthropology, University of Göttingen Göttingen Germany; ^2^ Institute for Medical Biometry and Bioinformatics, Heinrich Heine University Düsseldorf Düsseldorf Germany; ^3^ Wildlife Sciences University of Göttingen Göttingen Germany

**Keywords:** Alaudidae, breeding success, crop diversity, home range, nest success, winter cereals

## Abstract

The lack of suitable nesting sites is one key driver behind the farmland bird crisis in Europe. Winter cereals become impenetrable for ground‐breeding birds like the Eurasian Skylark (*Alauda arvensis*), curtailing breeding time. Stable Skylark populations depend on multiple breeding attempts per year; thus, the widespread cultivation of winter cereals has strongly contributed to their tremendous decline. Crop diversification is thought to be a potential measure to counteract this development. Therefore, we explored how individual Skylarks respond to the decreasing suitability of winter cereals as nesting habitats in heterogeneous but otherwise conventionally managed farmland. Our study focused on: (i) the degree to which Skylarks prematurely cease nesting activity, switch nesting habitats, or breed on linear structures like tramlines. Additionally, we analyzed: (ii) if nest success decreases throughout the breeding season and (iii) how often Skylarks make a successful breeding attempt per year. We radio‐tagged 28 adults in a German population during April 2018 and 2019, tracked half of them for more than 3 months, and measured their breeding success. Additionally, we monitored nests of untagged pairs, resulting in 96 nests found. None, except one tagged individual, stopped breeding activity before July 1st. Home ranges were mainly stable, but Skylarks switched nesting habitats away from winter cereals to crops like sugar beet or set‐aside. High‐risk nesting sites like corn and linear structures played a minor role in breeding. Overall, Mayfield logistic regressions revealed no seasonal decrease in nest success, and tagged Skylarks had sufficient time to make 1.5–1.8 breeding attempts, of which 0.8 were successful. We suggest that heterogeneous farmland in our study area, which enabled diversely composed home ranges, prevented a curtailment of the breeding season. Thus, our study reinforces the need for crop diversification which gives Skylarks a chance to survive in modern farmland.

## Graphical abstract

Dense‐growing crop vegetation in modern farmland is thought to curtail the potential time for breeding of Eurasian Skylarks (*Alauda arvensis*), thus contributing to the dramatic population decline in Skylarks in Europe. Contradicting this, we found no time constraints for successful reproduction in our Central European study population after following radio‐tagged Skylarks during the breeding season. Our findings are most likely a result of the heterogeneous composition of the study area, which guaranteed the constant availability of accessible and safe nesting habitats. Thus, our work reinforces the need for crop diversification that gives Skylarks a chance to survive in modern agricultural landscapes. Credits: Lisa Dumpe.

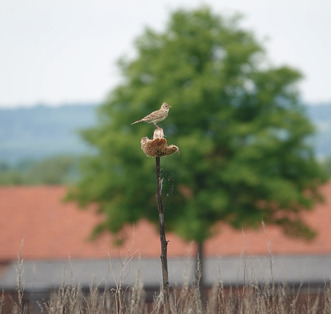

## 1 INTRODUCTION

Despite international agreement to halt biodiversity loss within the European Union (EU; European Commission, 2011), the tremendous declines in farmland birds as a consequence of agricultural intensification are still ongoing (European Environment Agency, 2020; Keller et al., 2020). Greening measurements of the Common Agricultural Policy (CAP) have failed to prevent further losses (Pe'er et al., 2017). Instead, policy‐driven increases in corn cultivation and decreases in the area of fallow land further worsened the situation (Busch et al., 2020; Tarjuelo et al., 2020; Traba & Morales, 2019). Butler et al. (2007) identified the loss of food and nesting habitats as key drivers underlying the biodiversity crisis in cropped farmland areas. Among the bird species that greatly suffered from a deficiency of nesting habitats caused by intensive farming is the Eurasian Skylark (*Alauda arvensis*; Donald, 2004; Hagist & Zellweger‐Fischer, 2020). Since 1980, the Skylark population of Europe has more than halved (PECBMS, 2022).

As ground‐nesting birds with multiple breeding attempts per year, Skylarks depend on vegetation that provides both sufficient nest cover and good accessibility throughout the breeding season (Donald, 2004; Jenny, 1990; Jeromin, 2002). In arable farmland, individual crops typically fulfill these conditions only within a certain period (Schläpfer, 1988; Wilson et al., 1997). This crop‐specific timeframe of suitability is of particular importance regarding cereals. More than 40% of arable land in the EU is cultivated with cereals (excluding corn and rice; Eurostat, 2022). Because of their prevalence, a high proportion of European Skylarks inhabit these cereals, so previous changes in cereal management are thought to have strongly influenced their population dynamics (Donald, 2004; Donald & Morris, 2005; Donald & Vickery, 2000). In particular, several studies suggest that the replacement of spring‐sown cereals with autumn‐sown cereals in many European regions heavily affected the breeding performance of Skylarks (Chamberlain et al., 1999; Donald & Vickery, 2000; Jenny, 1990; Siriwardena et al., 2001). Unlike spring‐sown cereals, autumn‐sown cereals have a dense sward structure that allows only one breeding attempt in the early breeding season (Chamberlain et al., 2000; Donald & Morris, 2005; Wilson et al., 1997). Therefore, Skylarks that initially nest in winter cereals have three different options to deal with this development.

First, individual breeding pairs can forgo further breeding attempts and prematurely cease nesting activity (Daunicht, 1998; Donald, 2004). Second, Skylarks can choose unvegetated tramlines within cereal fields or field edges as nesting sites later in the breeding season, which are easy access routes for predators, leading to high predation rates (Donald et al., 2002; Fischer et al., 2009; Püttmanns et al., 2021). Third, breeding pairs can build their nests in habitats with more accessible vegetation (Fischer et al., 2009; Ottens et al., 2013; Schläpfer, 1988). However, territorial shifts are often necessary to access alternative nesting habitats (Schläpfer, 2001) in response to increasingly homogenized farmland (Benton et al., 2003). Shifts were documented for individual breeding pairs (Jenny, 1990; Schläpfer, 1988) or concluded from seasonal shifts in habitat‐specific territory density (Eggers et al., 2011; Toepfer & Stubbe, 2001; Koleček et al., 2015). Furthermore, alternative nesting habitats in intensified agricultural landscapes are often unsuitable as in the case of corn due to the high predation risk (Praus & Weidinger, 2015) and grassland due to regular mowing (Ottens et al., 2013). Both the switch to accessible yet dangerous nesting habitats and the choice of linear structures as nesting sites would reduce the *nest success* of Skylarks (i.e., the success of individual nests, Ottens et al., 2016) during the breeding season. The potential time for successful breeding attempts is restricted to the early breeding season in all three options. This curtailment may be the main reason for the European decline. Two to three potentially successful breeding attempts per pair and year, depending on first‐year and adult survival rates, are probably essential for self‐sustaining populations (Wilson et al., 1997; Wolfenden & Peach, 2001).

One way to counteract a curtailment of the breeding season could be recovering habitat heterogeneity through crop diversification. Heterogeneous crop mosaics may supersede territory abandonments or shifts and enable multiple breeding attempts (Wilson et al., 1997). Several studies suggest crop diversification halts the decline in Skylarks, farmland birds in general, and the overall agrobiodiversity (Eraud & Boutin, 2002; Šálek et al., 2021; Schläpfer, 1988; Sirami et al., 2019; Tscharntke et al., 2021). To our knowledge, however, it remains untested if habitat heterogeneity alone is a sufficient conservation measure in an intensified agricultural landscape including, e.g., a high input of chemicals.

Therefore, we aimed to investigate how individual Skylarks respond to the decreasing suitability of winter cereals as nesting habitats in heterogeneous but otherwise conventionally managed farmland. We analyzed: (i) the degree to which Skylarks prematurely cease nesting activity, breed on linear structures of winter cereals, or switch nesting habitats when vegetation becomes impenetrable. Moreover, we wanted to know if successful breeding is restricted to the early breeding season. In this context, we analyzed: (ii) if nest success decreases throughout the breeding season, and (iii) how often Skylarks make a successful breeding attempt per year, here defined as *breeding success*. Thus, we tracked radio‐tagged Skylarks throughout the breeding season, measured the breeding success, and corroborated our findings with nesting data of untagged pairs.

## 2 METHODS

### 2.1 Study area

**FIGURE 1:**
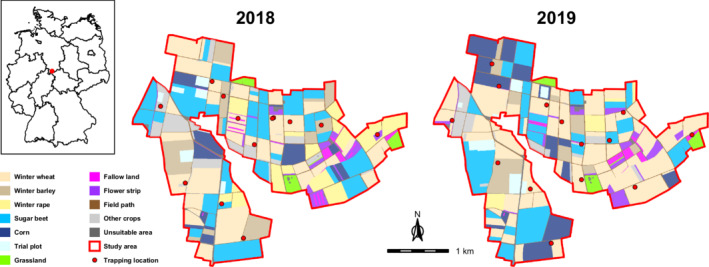
Location of the study area (red dot) within Germany (upper left corner) and its composition in 2018 and 2019. The same map design as in Püttmanns et al. (2022) was chosen to facilitate comparison. Other crops = asparagus, broad bean, clover, cup plant, potato, strawberry, summer barley, summer wheat, winter rye, and winter triticale; Unsuitable area = building, company site, highway, water body, and wood. Trapping locations are shown for sites where at least one Skylark was caught during April. Shapefiles of individual fields were provided by the *Servicezentrum Landentwicklung und Agrarförderung*, shapefiles of Germany, and its federal states by DIVA‐GIS (2021).

The study area (8.1 km^2^) is located in the south of Göttingen, Germany (N51° 29.631, E9° 56.595). On a national scale, the regional climate is comparatively dry (mean annual temperature: 8.7°C, mean annual total precipitation: 644.9 mm; Vohl, 2020). Fieldwork was carried out between April and August from 2018 to 2019. Skylark densities varied between three to four territories per 10 ha (estimations based on Langer 2017 and Meineke 2020, unpublished data). Farmland dominated the study site with 85.8% cropland and only 1.9% permanent grassland. Fields under organic farming made up 3.8%. The mean size of arable fields was 4.8 ha. Although winter cereals were the most cultivated crops in 2018 and 2019 (winter wheat: 34.5% of the whole study area averaged over both years; winter barley: 8.2%), the study site lacked extensive monocultures. Instead, sugar beet (21.2%), corn (9.6%), winter rape (6.9%), and other crops (e.g., asparagus, broad bean, clover, strawberry, and summer wheat: each ≤1.3%) were often cultivated next to winter cereals. Trial plots (2.4%) of the Faculty of Agricultural Sciences from the University of Göttingen, sown flower strips (3.0%), and fallow land (1.3%), which were mainly established in the framework of the PARTRIDGE conservation project (PARTRIDGE, 2021), further enriched the composition of habitats. The local network of field paths covered an area of 2.9% with a total length of approx. 30.2 km. Structures that Skylarks generally avoid, like buildings, hedgerows, or woods, were rare (Figure 1).

### 2.2 Bird data

#### 2.2.1 Radio‐tracking

At the beginning of the breeding season in April 2018 and 2019, we caught 14 Skylarks per year using mist nets. The netting occurred at dawn or dusk within areas of high territorial activity across the study area (Figure 1). We used playback of the male song as a luring device. Birds were ringed and equipped with a radio‐tag (2018: standard pulse transmitter, 1.0–1.2 g, JDJC Corp., USA; 2019: PIP Ag392 for males, PIP Ag376 for females, 0.8 g to 1.2 g, Lotek, UK) that equaled 3–4% of the body weight. We glued the transmitter on a piece of cloth to the shortened back feathers. For sexing individuals, we used the wing length, as females have shorter wings, revealing a total of 23 tagged males and 5 females, including one pair. Later, field observations of sex‐typical behavior (e.g., males in song flight) confirmed the sexing in all cases. Following their release, individuals were tracked twice a day for 5 days a week using a three‐element folding Yagi antenna (F150‐3FB, AF Antronics Inc., USA) and a telemetry receiver (R‐1000, 148–174 MHz, Communications Specialists Inc., USA). In 37% of the localizations, we directly observed the tagged bird. All other positions were determined by triangulation. The mean time interval between two localizations of the same day was 3.6 h with a minimum of 1 h. Radio‐tracking lasted until the tag fell off (21 cases), the signal was lost (5), or the bird was predated (2). On average, we tracked the Skylarks for 79 days, with 13 birds covering the whole Central European breeding season from mid‐April to the end of July (Glutz von Blotzheim & Bauer, 1985).

We caught an additional three Skylarks (two males, one female) in June 2018 to compensate for early tag losses. Two of these were partners of individuals that had recently lost their transmitter, so we could still make inferences on home range changes and the individual breeding success. Both were caught with mist nets erected above their nests following the methods of Ottens et al. (2016). We tracked the additionally captured birds as described above until the loss of the transmitter (two) or predation (one).

#### 2.2.2 Nest monitoring

We had to document all successful breeding attempts of radio‐tagged individuals during a single breeding season to measure the breeding success of Skylarks. Using a hide, such as a camouflaged tent or car, we systematically observed the behavioral events around the most recent localizations of individuals for several hours per week. When we noticed clear indications of a nest (nest building, female returning to the nest for incubation, and feeding of chicks), we searched for the nest in the focal area of breeding activity. Rope dragging to flush incubating females, a thermal binocular (Pulsar Accolade XQ 38), and the radio‐tracking itself, in case of tagged females sitting on the nest, further assisted the nest search. We confirmed the tagged individual as a parent of the nest by checking the strength of the radio‐signal when the bird approached the nest or by visually observing the attached transmitter. Unfortunately, four transmitters fell off, and one tag stopped working shortly before we found a nest close to the last recent localizations of the respective individual. Therefore, we confirmed with binoculars if a bird that approached the nest had a non‐working transmitter on its back or a ring on its leg, strongly suggesting the tracked bird was a parent. We also searched for nests of untagged individuals across the study area from April to August to analyze seasonal changes in nest success (i.e., the success of single nests) based on a larger dataset. Our efforts resulted in a total of 96 nests, 31 of which were breeding attempts of radio‐tagged Skylarks. The contents of nests were checked approximately every 3rd day to document the nest outcome. The distinction between predation and success (i.e., chicks had left the nest) was usually simple due to visual and/or acoustic cues (predation: messy nesting material, remains of eggs, and injured dead chicks; success: warning or food‐carrying adults, cheeping chicks, or their feces close to the empty nest). In case of no apparent signs, we counted empty nests whose chicks were younger than the 7th day as predated (Donald et al., 2002). The state of physical development was used for aging and determined according to Pätzold (1983). Nests with eggs were counted as abandoned if there was no observed activity at the nest and chicks had not hatched within 2 weeks after the nest was found, as incubation lasts 13 days at most (Donald, 2004). We also documented the clutch size, nesting habitat, and the distance to adjacent tramlines and field edges for nests in winter cereals. To back‐calculate the date of the first egg, we assumed a laying rate of one egg per day, 10 days for incubation (excluding the day of laying the last egg and day of hatching), and synchronous hatching (Donald, 2004; Donald et al., 2002). In the case of 14 nests that were predated before chicks were found at a nest control, we could not consider the chick age for our calculations. Then, we chose the midpoint between the earliest and latest possible first egg date, which depended on the number of days a nest was known to be active.

### 2.3 Vegetation data

To discuss potential territory abandonments or home range shifts in the context of crop development, we also collected vegetation data. Crop openness and height of seven winter wheat fields in 2018 and a maximum of eight fields in 2019 were measured in intervals of, on average, 10 days. Each field assessed was regularly used by one of the radio‐tagged Skylarks during their first days of radio‐tracking. As a proxy for openness, we estimated the fractional vegetation cover following the descriptions in Püttmanns et al. (2022). Crop height was defined as the top of the crop canopy level and measured with a folding ruler. Measurements started on May 9th in 2018 and April 19th in 2019 and ended with the wheat harvest.

### 2.4 Data analysis

#### 2.4.1 Individual breeding activity

To begin analyzing Skylark behavior in denser winter cereal vegetation, we checked if tagged individuals ceased breeding activity clearly before the end of the breeding season. We defined the absence of breeding activity before July 1st as premature termination, as May and June are primarily when most breeding attempts occur in Central Europe (Donald, 2004). Signs of breeding activity were (i) active nests of respective individuals and (ii) the defense of a territory which was inferred from observations of song flights or antagonistic behavior against neighboring Skylarks (Schläpfer, 1988). Although single Skylarks or nonbreeding pairs can also defend a territory (Delius, 1965; Wilson et al., 1997), these birds abandon their territories in the course of the breeding season (Delius, 1965). Therefore, holding a territory indicates at least a high interest in breeding. We only considered 15 males that could be tracked until the end of July for our analysis.

Moreover, we investigated the onset of breeding attempts during the breeding season. We used a mixed‐effect logistic regression model (GLMM) to test whether radio‐tagged Skylarks were less likely to start a breeding attempt later in the breeding season. First, we calculated the 2.5 to 97.5 percentile range of first egg dates based on all 96 nests found. Only nests of tagged individuals with first egg dates within this range, i.e., between April 14th and July 10th, were included in the model. We further subdivided the period into equal intervals of 22 days (phase 1: April 14th to May 5th; phase 2: May 6th to May 27th; phase 3: May 28th to June 18th; and phase 4: June 19th to July 10th). An interval of 22 days was chosen, as it represents the average duration of a complete breeding cycle, with 14 days of the egg‐laying and incubation stage and 8 days of the nestling stage (Praus et al., 2014). The phase was used as a categorical predictor for modeling, while the onset of a breeding attempt (yes/no) was taken as a binary dependent variable. Additionally, the year was included as a fixed effect and the individual as a random effect:

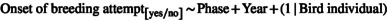

The phase of a radio‐tagged Skylark was only considered in the model when the respective individual (i) was not involved in an active breeding attempt for at least 4 days, as this is the minimum time for a female to lay the first egg of a new attempt (Delius, 1965; Donald, 2004), and (ii) showed breeding activity as defined above. For female Skylarks that do not defend territories by song flights, we interpreted mate guarding by males as an additional sign of breeding activity (Donald, 2004). Overall, 30 tagged individuals, including three pairs, were used for modeling. Skylarks were equally weighted in the analysis, with pairs defined as a single unit.

#### 2.4.2 Home range shifts

Before examining the choice of nesting habitats during the breeding season, we identified radio‐tagged birds that had shifted their home ranges. First, we digitalized the localizations of all tracked individuals in *ArcGIS* (version 10.3.1; Esri Inc. 1999–2015; WGS 84/UTM zone 32 N). The underlying map of the study area was based on field shapefiles provided by the *Servicezentrum Landentwicklung und Agrarförderung*. Then, we used *R* (version 4.0.3, R Core Team, 2020) to perform a modified approach of Filla et al. (2017) to detect home range shifts. Instead of analyzing the size of minimum convex polygons (MCP95, Mohr, 1947) over time, we examined centroid shifts of tracking data, allowing for the combination of data from a tagged pair (see below).

**FIGURE A1:**
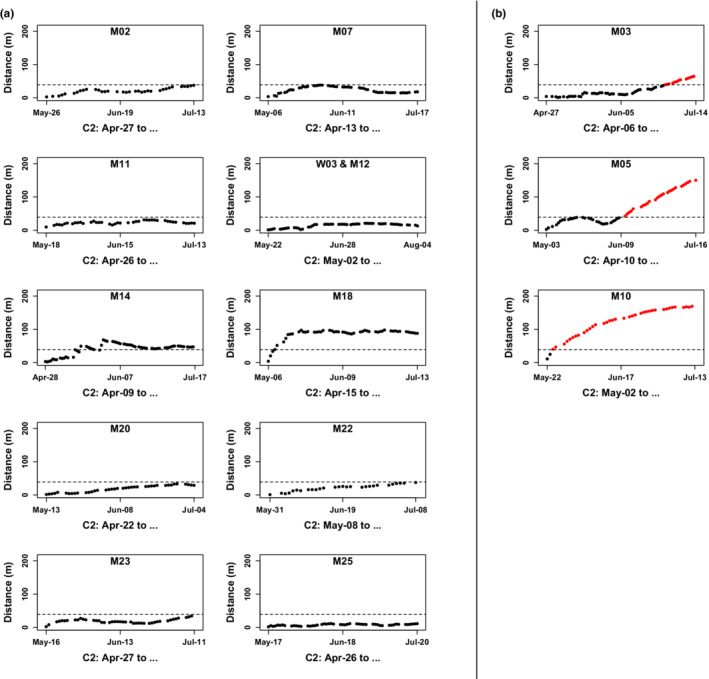
Distances between the centroid of the original home range (C1) of radio‐tagged Skylarks (*Alauda arvensis*) and the centroids based on stepwise addition of daily localizations over time (C2). Individuals were grouped according to their home range maintenance (a) or shift (b) during the breeding season. Home range shifts were defined as a steady increase in distance exceeding the dashed line at 39 m (red points). The sharp increase in distance by M18 that levels off afterwards indicates the bird expanded the home range early in the breeding season but still used the original area.

The centroid of an individual's first 30 localizations after the onset of breeding activity (see Section 2.4, 2.4.1) was defined as the centroid of the original home range. We chose 30 localizations to reach the minimum number of 20 to 30 for representing a Skylark home range (discussed in Jeromin, 2002) without overestimating its size due to possible early home range shifts. Next, we added the two localizations of the subsequent tracking day, re‐calculated the centroid of all 32 data points, and measured the distance between the new centroid and the original centroid. This was repeated until we reached the date with the last sign of breeding activity. Plotting the centroid distances over time revealed the home range shifts as they became apparent with a continual increase in distance (Figure A1). This pattern could only occur when a bird settled in a new area and was almost exclusively located there each additional tracking day, inducing a continuous centroid shift. Preliminary tests showed that discrepancies between documented localizations and the “true” position (the exact coordinates of the transmitter position) had a 95th percentile of 39 m, so we considered a steady increase beyond this distance as a true home range shift. Only 12 birds tagged during April and with breeding activity beyond July 1st were considered. Additionally, we combined tracking data from a female (W03), which lost its transmitter in June, with the tracking data of its partner (M12), which was radio‐tagged immediately after the loss. Although partners may have home ranges of different sizes (Jeromin, 2002), we did not expect an influence on our analysis of centroids.

**FIGURE A2:**
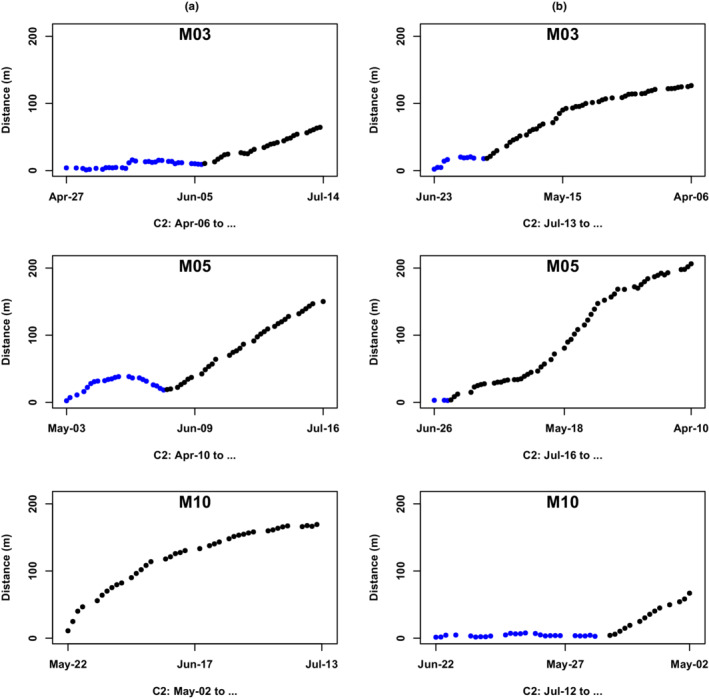
Assignment of localizations of radio‐tagged Skylarks (*Alauda arvensis*) that shifted their home ranges to the early (a) or late MCP95 (b). Localizations of dates before the beginning of the continual increase were used to calculate the early/late MCP95 (blue points). The first 30 localizations used to determine the centroid of the original home range C1 were always considered for the respective calculations.

To deduce if detected home range shifts were related to the decreasing habitat suitability of winter cereals, we compared the composition of home ranges between birds with and without home range shifts. For tracked Skylarks with stable home ranges (i.e., without clear centroid shifts), we used all localizations within the individual period of breeding activity to calculate the composition of the MCP95s. Despite its limitations, the boundaries of MCPs are adequate to outline the available habitat for individuals (Horne et al., 2009; Horne et al., 2020). For birds that shifted their home range, we calculated the composition of an early and a late MCP95. To assign localizations to the early MCP95, we examined the original plots of centroid distances. All localizations of dates before the beginning of the continual increase (i.e., before the onset of the home range shift) were used to calculate the early MCP95 (Figure A2a). To assign late MCP95 localizations, we reran a reverse analysis of centroid shifts: the centroid of an individual's last 30 localizations before breeding activity ended was defined as the original home range. We added the two localizations of the preceding tracking day, recalculated the centroid, and measured the distance between the new and the original centroid until we reached the first breeding activity date. Localizations of dates before the beginning of the continual increase were then used to calculate the late MCP95 (Figure A2b). All localizations of dates not assigned to either the early or the late MCP95 were defined as transitional habitat use and excluded from our analysis.

#### 2.4.3 Nest habitats, nest success, and breeding success

After grouping radio‐tagged Skylarks with sufficient tracking data into groups that prematurely ceased nesting activity, shifted their home range later in the breeding season, or kept their home range, we analyzed the choice of nesting habitats over time. A Fisher's exact test was conducted to test if the use of nesting habitats generally differed throughout the breeding season. For individuals nesting in cereals, we checked if later breeding attempts were positioned closer to tramlines or field edges than earlier attempts. Moreover, we compared the pattern of nesting habitats of radio‐tagged Skylarks with the nesting site phenology of all nests found.

Similarly, we examined if successful nests of tagged individuals were more frequent in the early breeding season and evaluated our conclusions based on an analysis of the daily nest survival (DNS) that considered the dataset of all nests found. The program MARK (White & Burnham, 2009) via the *RMark* package (Laake, 2013) was used to perform Mayfield logistic regressions (Hazler, 2004). A binary categorization of nest outcome (1 = success; 0 = failure) was used as a response variable. We built two types of models: a *habitat model* and a *seasonal model*. In the habitat model, we included the nesting habitat as a nominal predictor variable to analyze if certain habitat types were more dangerous than others. Nesting sites were grouped into the habitat categories: *winter cereals*, *sugar beet*, *corn, other summer crops* (broad bean, strawberry, and summer wheat), *mowed areas* (clover and grassy area of trial plots), and *set‐aside* (fallow land and flower strips). Only nests positioned farther than 0.5 m from the next field edge or tramline were included in the category *winter cereals*. Nests in winter cereals closer than/at 0.5 m distance were sorted into the additional category *linear structures* according to Püttmanns et al. (2021). In the *seasonal model*, we used the day of the first egg (day 1: April 11th as our earliest calculated first egg date) as a predictor to test if the DNS, and thus the overall nest success, decreased during the breeding season. The year and the radio‐tagging (yes/no) were included in both models as fixed effects.

Habitat model:

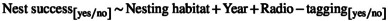




Seasonal Model:

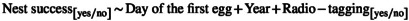

Effects of the habitat diversity surrounding nests and the distance to the next vertical structure (e.g., hedgerow and tree) were not included in the models. Predator activity is primarily driven by the vegetation type at a specific site and less so by habitat diversity (Laux et al., 2022). Additionally, Skylarks avoid vertical structures when choosing a nest location (Donald, 2004), so the distance to those structures in our study area was large and uninformative (mean distance between nests and vertical structures: 129 m ± 77 standard deviation). Furthermore, we did not adjust to changing weather conditions. We collected our data in 2 years of extremely dry weather (Deutscher Wetterdienst, 2021; Zscheischler & Fischer, 2020) without detrimental rainfall that may have affected nest outcome (Donald et al., 2001). To calculate the chance for nests to survive a complete breeding cycle, we raised the DNS to the power of 22, representing the average duration of a full breeding cycle (see Section 2.4.1). All four abandoned nests were not included in our analysis due to the difficulty of defining the date of abandonment (see Section 2.2.2). A further eight nests were not considered because the nest became inactive shortly before the find (six), or the nest outcome was influenced by human intervention (two).

We calculated the average number of successful breeding attempts together with the average number of chicks that had left the nest per radio‐tagged individual (or pair) and breeding season to determine breeding success. Only the 17 birds tagged during April, including two pairs, with tracking data until the end of July, were considered for the calculations.

## 3 RESULTS

### 3.1 Individual breeding activity

**FIGURE 2:**
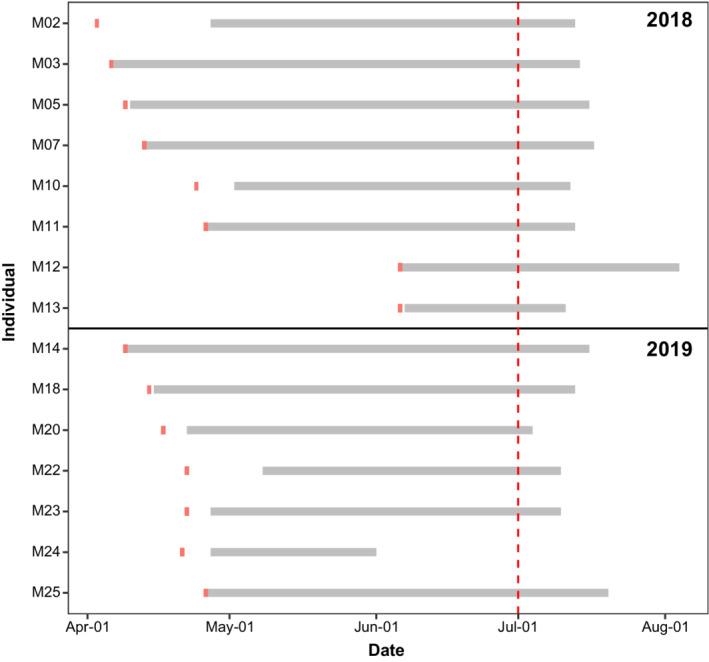
Duration of breeding activity of 15 radio‐tagged male Skylarks (*Alauda arvensis*). Gray bars cover the period between the first and the last sign of breeding activity. Red markers indicate the start of radio‐tracking. The absence of breeding activity before July 1st, indicated by the red dashed line, was defined as premature termination.

Only 1 of the 15 male Skylarks (M24) tracked until the end of July showed no sign of breeding activity from the beginning of June onwards. For 12 males (80%), we documented the last sign of breeding activity within the 5 days around July 15th (Figure 2).

**FIGURE 3:**
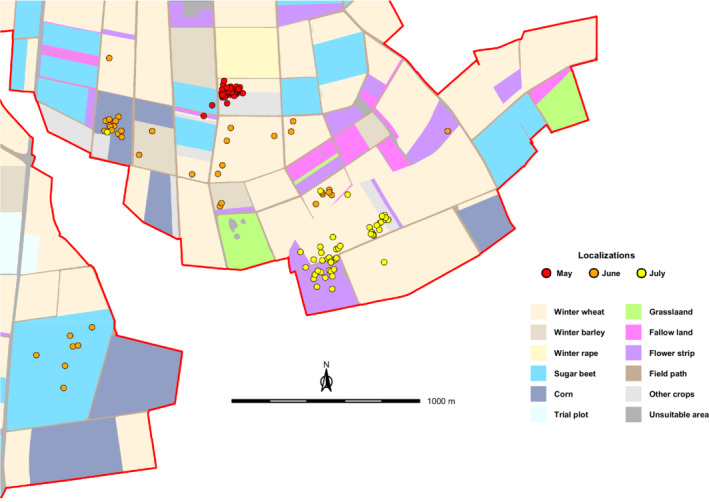
Localizations of Skylark (*Alauda arvensis*) male M24 within the study area (red line) from May to July 2019. Other crops = asparagus, broad bean, clover, cup plant, potato, strawberry, summer barley, summer wheat, winter rye, and winter triticale; Unsuitable area = building, company site, highway, water body, and wood.

Male M24 had an active nest until the end of May 2019. After nest predation around June 1st, it started to roam across the eastern half of the study site. From July on, localizations were again concentrated in a distinct area, at which M24 was regularly accompanied by a second bird. A restart of breeding activity could not be observed (Figure 3).

Our GLMM revealed that the probability of radio‐tagged Skylarks starting a breeding attempt during the second phase (May 6th to May 27th) did not differ significantly from the probability of starting a breeding attempt during any other phase. The year had no significant effect. (Table 1).

**TABLE 1:** Summary of the mixed‐effect logistic regression model describing the effect of the ongoing breeding season on the probability for radio‐tagged Skylarks (*Alauda arvensis*) to start a breeding attempt. Phase 2 (May 6th to May 27th) was set as the reference time because it covers the beginning of the main breeding season in Central Europe (Donald, 2004). Phase 1 = April 14th to May 5th; Phase 3 = May 28th to June 18th; Phase 4: June 19th to July 10th. The estimates (Est.), standard errors (Std. error), lower 95% confidence intervals (Lower 95% CI), upper 95% confidence intervals (Upper 95% CI), *z*‐values (z), and *p*‐values (*p*) are given for each fixed effect. The standard deviation of the random effect (Bird ID) was ±0.366. *n* = 77 phases of 30 radio‐tagged Skylarks, including 3 pairs.

Fixed effect	Est.	Std. error	Lower 95% CI	Upper 95% CI	*z*	*p*
Intercept	−0.173	0.515	−1.295	0.841	−0.336	.737
Phase 1	−0.990	0.632	−2.311	0.220	−1.566	.117
Phase 3	0.382	0.725	−1.017	1.895	0.527	.598
Phase 4	−0.583	0.756	−2.148	0.921	−0.771	.441
Year 2019	−0.147	0.523	−1.276	0.958	−0.280	.779

### 3.2 Home range shifts

**FIGURE A3:**
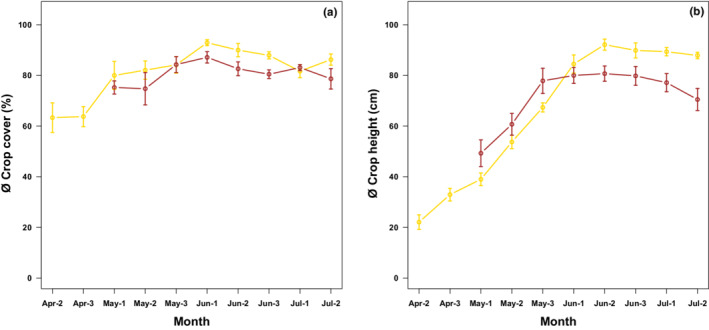
Average (±SE) crop cover (a), and crop height (b) of winter wheat fields in the course of the breeding season. Brown line = 2018; yellow line = 2019. Month‐1 = 8th–10th; Month‐2 = 18th–20th; Month‐3 = 29th–30th. Sample sizes differ due to earlier harvest of single fields in 2018 and because not all fields were always measured in 2019: *n* (2018; May‐1–Jul‐1) = 7; *n* (2018; Jul‐2 = 5); *n* (2019; Apr‐3; May‐2–Jun‐1; Jun‐3–Jul‐2) = 8; *n* (2019; May‐1; Jun‐2) = 7; *n* (2019; Apr‐2) = 6.

Nine of the 12 males tagged during April and with tracking data beyond July 1st, as well as the Skylark pair with combined tracking data, stayed in the same home range throughout the breeding season. One of the nine males (M18) expanded its home range in early May. We documented clear home range shifts for three males (Figure A1). The shifting started between the end of May and the beginning of June (Figure A2a). During this time, crop cover of winter cereals exceeded 80% and crop height 70 cm. (Figure A3). On average, the centroids between the early and the late home range were 218 m apart (Table 2).

**TABLE 2:** Overview of the three detected home range shifts by radio‐tagged Skylarks (*Alauda arvensis*). The distance between the centroids of the early and the late MCP95 was defined as the distance between the home ranges.

Bird ID	Onset of home range shift	Distance between home ranges (m)
M03	June 8th, 2018	147
M05	June 1st, 2018	310
M10	May 22nd, 2018	197
Mean	May 31st, 2018	218

**FIGURE 4:**
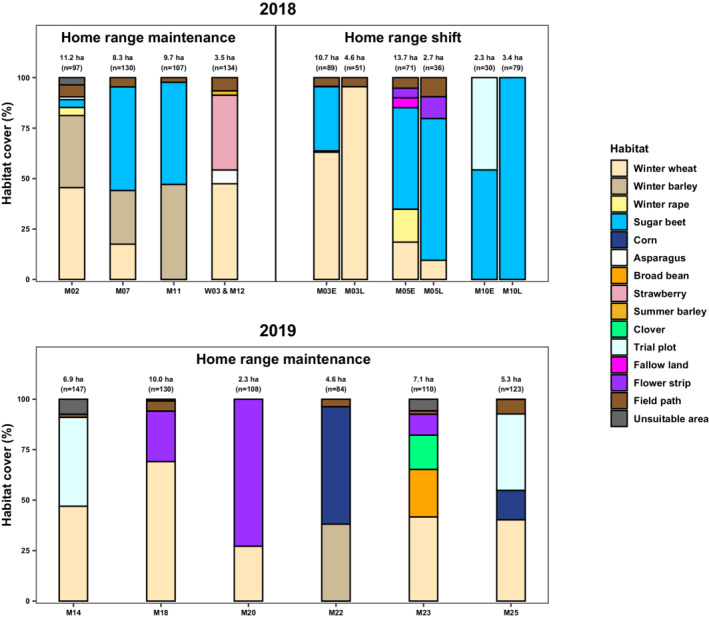
Home range composition of radio‐tagged Skylarks (*Alauda arvensis*). E = composition of the early MCP95 before the shift; L = composition of the late MCP95 after the shift. Unsuitable area = building, company site, highway, water body, and wood. Sample sizes (*n*) refer to the number of points used to calculate the MCP95.

All except two of the analyzed home ranges were composed of at least winter cereals and a summer crop, trial plot, or flower strip (Figure 4). The average proportion of winter cereals in the early home ranges of the three shifting individuals was 27.20% (±32.44). Skylarks that kept their home range during the breeding season had a higher winter cereal proportion of 48.31% (±15.62). The early home range of male M03 had a smaller proportion of winter cereals than its late home range (Figure 4).

### 3.3 Nesting habitats

Of the 96 active nests found during data collection in 2018 and 2019, 49 nests were initiated in winter cereals with 8 nests close to linear structures, 18 in sugar beet, and 13 in set‐aside. A further seven nests were built in less common summer crops (broad bean: four, summer wheat: two, and strawberry: one), five on mowed areas, and four in corn.

**FIGURE 5:**
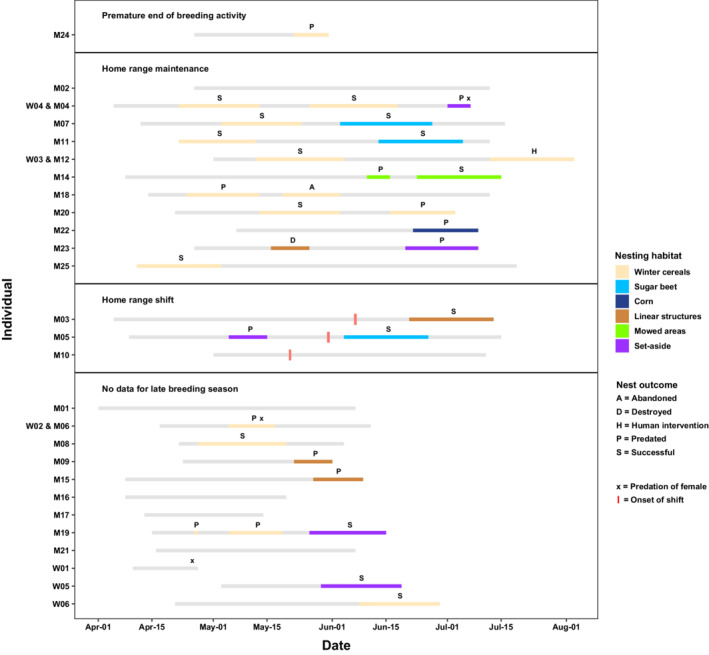
Overview of nest habitats, nest success (i.e., the survival of individual nests), and breeding success (i.e., the number of successful breeding attempts) of radio‐tagged Skylarks (*Alauda arvensis*). Gray bars cover the period of breeding activity without active nests. The bars of nesting habitats cover the period during which a nest was active. Birds without data for the late breeding season were predated, lost the radio‐tag, or the tag stopped working. Destroyed = destroyed by agricultural practice; human intervention = intervention through which a parcel of cereals with the nest in the center was not harvested.

Among radio‐tagged Skylarks, winter cereals were the dominating nest habitat during the early breeding season. Eleven of the 12 breeding attempts that began before the second half of May were found in winter cereals (Figure 5). From then onwards, other nest habitats like set‐aside (four nests), sugar beet (three), mowed areas (two), and corn (one) outnumbered the use of winter cereals (six). Moreover, the four breeding attempts on linear structures in winter cereal fields were not started before the second half of May (Figure 5).

**FIGURE 6:**
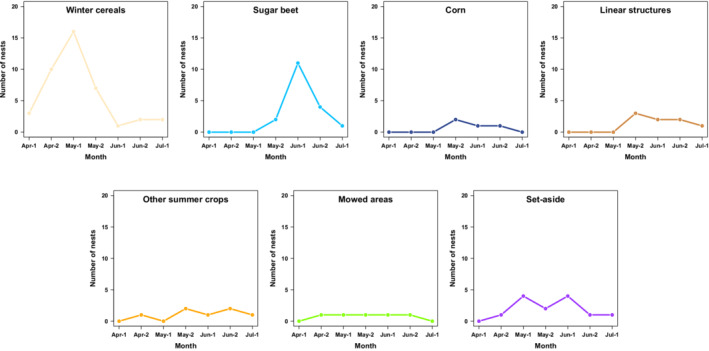
Nesting habitats of Skylarks (*Alauda arvensis*) for the duration of the breeding season. Month 1 = month 1st–15th; Month 2 = month 16th–30th/31st. Nests were assigned according to their first egg dates. *n* = 96 (including the nests of radio‐tagged individuals).

When considering all nests found, the use of nesting habitats differed across the breeding season according to Fisher's exact test (*p* < .001). Winter cereals and sugar beet, the two dominating crop types in the study area, were also the two most important nest habitats with a clear time shift in use (Figure 6). The use of winter cereals as nest habitat reached its peak during the first half of May (16 nests) and then strongly decreased, whereas the use of sugar beet reached its peak during the first half of June (11). Corn and linear structures were less frequently used (each ≤3 nests per half of a month) and did not appear as nesting habitats before the second half of May. Nests in other summer crops, mowed areas, and set‐aside were found consistently throughout the breeding season, but to a smaller extent (each ≤4 nests per half of a month; Figure 6).

### 3.4 Nest success

Considering all 96 nests, 68 were successful, 20 were predated, 4 were abandoned, 1 was destroyed by agricultural practice, and 1 nest was unsuccessful because the chicks failed to hatch. In two cases, the nest outcome was influenced by human intervention, as a parcel of cereals with the nest in the center was not harvested.

Nest success of radio‐tagged Skylarks did not decrease during the breeding season. Consequently, 50% of nests with first egg dates before June 1st were successful, and 50% of nests were successful after this date (Figure 5).

Mayfield logistic regressions revealed no significant effect of the time of the breeding season on the DNS. However, the habitat of nesting sites affected nest success. Corn and linear structures as nesting habitats reduced the DNS compared to winter cereals with statistical significance. Radio‐tagging of Skylarks likewise had a negative effect. Other nest habitats and the year had no statistically significant influence on the DNS (Tables 3 and 4).

**TABLE 3:** Summary of the Mayfield logistic regression describing the effect of different nest habitats on daily nest survival of Skylark (*Alauda arvensis*) nests. Winter cereals were set as reference habitats. The estimates (Est.), standard errors (Std. error), lower 95% confidence intervals (Lower 95% CI), upper 95% confidence intervals (Upper 95% CI), and the levels of significance (*p*) are given for each fixed effect. *n* (nests) = 84.

Fixed effect	Est.	Std. error	Lower 95% CI	Upper 95% CI	*p*
Intercept	4.672	0.667	3.366	5.979	<.001
Sugar beet	0.056	1.122	−2.142	2.254	.960
Corn	−2.937	0.984	−4.865	−1.010	.003
Other summer crops	−0.846	1.161	−3.121	1.429	.466
Mowed areas	−1.643	0.899	−3.404	0.118	.067
Set‐aside	−0.593	0.745	−2.053	0.867	.426
Linear structures	−2.009	0.687	−3.356	−0.663	.003
Year: 2019	−0.289	0.564	−1.393	0.816	.608
Radio‐tagging: yes	−1.205	0.556	−2.294	−0.116	.030

**TABLE 4:** Summary of the Mayfield logistic regression describing the effect of the ongoing breeding season on daily nest survival of Skylark (*Alauda arvensis*) nests. The estimates (Est.), standard errors (Std. error), lower 95% confidence intervals (Lower 95% CI), upper 95% confidence intervals (Upper 95% CI), and the levels of significance (*p*) are given for each fixed effect. *n* (nests) = 84.

Fixed effect	Est.	Std. error	Lower 95% CI	Upper 95% CI	*p*
Intercept	4.362	0.647	3.095	5.630	<.001
Day of first egg	−0.006	0.011	−0.028	0.017	.601
Year: 2019	−0.522	0.508	−1.517	0.473	.304
Radio‐tagging: yes	−0.979	0.460	−1.882	−0.077	.033

Overall, the average DNS of the habitat model was 0.9734 (standard error = ±0.0080; lower 95% confidence interval = 0.9524; upper 95% confidence interval = 0.9853) with a 55.29% (SE = ±9.99; LCI = 35.54; UCI = 73.51) chance for nest survival. According to the seasonal model, the average DNS was 0.9707 (SE = ±0.0075; LCI = 0.9516; UCI = 0.9823) with a 51.93% (SE = ±8.89; LCI = 34.65; UCI = 68.77) chance for nests to survive a complete breeding cycle.

### 3.5 Breeding attempts and breeding success

On average, we documented 1.53 (±0.83 standard deviation) breeding attempts per radio‐tagged individual or pair and year. Most birds made two breeding attempts (seven tagged individuals and one tagged pair), followed by individuals with one breeding attempt (four) or none (two). For one pair, we documented a total of three breeding attempts (Figure 5).

Of all breeding attempts, 0.79 (±0.80) were successful, producing an average of 2.38 (±2.79) chicks that left the nest. In 2018, the number of successful breeding attempts was 2.7 times higher, and the number of chicks was 3.4 times higher compared to 2019 (Table 5).

**TABLE 5:** Average breeding success (± standard deviation) of radio‐tagged Skylarks (*Alauda arvensis*) per breeding pair and year. Only tagged individuals/pairs that were tracked beyond July 1st were considered for calculations.

Year	*n*	No. breeding attempts	No. successful breeding attempts	No. of chicks per pair and year
2018	8	1.50 (±1.07)	1.14 (±0.90)	3.83 (±3.37)
2019	7	1.57 (±0.53)	0.43 (±0.53)	1.14 (±1.46)
2018 & 2019	15	1.53 (±0.83)	0.79 (±0.80)	2.38 (±2.79)

## 4 DISCUSSION

Despite winter cereals being the dominant crop in our study area, we found no temporal restriction of successful breeding attempts to the early breeding season of Skylarks. Neither the nest success decreased over time, nor was the breeding activity prematurely terminated. Only one radio‐tagged male abandoned its territory shortly after its nest had been predated. The male eventually found companionship with a second bird after roaming through large parts of the study area. This behavior resembled Delius' (1965) description of wandering non‐breeders searching for opportunities to replace territorial Skylarks. Former breeders that turn into floaters (see Penteriani et al., 2011) after nest and probably mate loss are already known from other bird species (e.g., from the middle‐spotted woodpecker *Dendrocoptes medius*, Robles & Ciudad, 2020). Thus, we assert that territorial abandonment was not motivated by the cessation of breeding activity but by searching for new breeding opportunities after nest predation and potential predation of the mate.

Instead of suggesting premature termination of the breeding season, our results support earlier findings describing shifts in nesting sites when winter cereal vegetation becomes denser (Fischer et al., 2009; Ottens et al., 2013; Schläpfer, 1988). As indicated by our radio‐tagged Skylarks, shifting of nesting habitats was not primarily a result of home range shifts as in other studies (Eggers et al., 2011; Jenny, 1990; Koleček et al., 2015; Schläpfer, 1988) but occurred within the original home range. Additionally, we do not think that the 3 of 13 Skylarks that shifted their home range were motivated by sward development. Although the onset of shifting coincided with winter cereals exceeding the typical suitable nesting vegetation height of 55–60 cm (Figure A3; Donald et al., 2002), home ranges of shifting individuals were not characterized by high winter cereal proportions. Moreover, in one case, the proportion of winter cereals was even higher after the shift. The respective individual chose a tramline for nesting in its new home range, which was an easily available micro‐habitat before the shift. We assume that home range shifts were triggered by a lack of breeding success, as we could not document a successful breeding attempt of any shifting individual during the early breeding season. Schläpfer (1988) already relates territorial stability in Skylarks to breeding success, and Hiron et al. (2012) suggest nest failure is behind the seasonal decline in Skylarks in winter cereals. Additionally, nest site shifts after breeding failure are reported for several other bird species and summarized as the *win–stay: lose–switch rule* (e.g., Chalfoun & Martin, 2010; Kearns & Rodewald, 2013). Nevertheless, our small sample size impedes safe conclusions except that home range shifts did not frequently occur among radio‐tagged Skylarks.

From the end of May onwards, we confirmed both linear structures of winter cereal fields (Donald et al., 2002; Donald & Vickery, 2000) and openly structured crops like corn (Schläpfer, 1988), as nesting habitats of tagged individuals. Furthermore, our data confirm the negative influence of closeness to linear structures (Donald et al., 2002; Fischer et al., 2009; Püttmanns et al., 2021) and nesting in corn (Praus & Weidinger, 2015) on nest success. Nevertheless, no indications of a seasonal decrease in nest success were found, which can be explained by the dominant use of sugar beet and the minor role of linear structures and corn during the late breeding season. Jenny (1990) found few nests in sugar beet, but these had the highest nest success compared to all other crops. According to our Mayfield logistic regressions, sugar beet as a breeding habitat had no negative effect on the DNS compared to winter cereals. Similarly, set‐aside (including flower strips) was not a particularly high‐risk nesting habitat. Three radio‐tagged Skylarks used set‐aside for their second or third breeding attempt, and overall, it was consistently used as a nesting habitat. Thus, our findings emphasize the value of uncropped land, which is a common measure to support farmland bird populations (Meichtry‐Stier et al., 2018; PARTRIDGE, 2021; Schmidt et al., 2022). The small proportion of grassland in our study area likely had positive effects on nest survival, as it reduced breeding opportunities in this high‐risk habitat (Kuiper et al., 2015; Ottens et al., 2013; Ottens et al., 2016). Our calculated nest success of ca. 52–55% is, to our knowledge, the highest value reported for breeding Skylarks from farmland thus far (compilation in Praus et al., 2014). We are confident that these results were not strongly biased by selective nest searching. Either we searched for nests after behavioral observations (e.g., collecting nesting material or food) that were primarily independent of the nesting site, or we focused on radio‐tagged Skylarks and their neighboring territories.

The negative effect of the radio‐tagging itself on the DNS could be traced back to the intensive observations of tagged individuals. This may have led to an increase in documented nesting attempts lost early after initiation. Additionally, our regular presence close to/within home ranges through tracking, observation, and nest search might have posed a disturbance, reducing energetic and time capacities for nest guarding. It is also possible that the tag increased the predation risk for incubating females, as three of six tagged females were predated: two during incubation. Both times feather remains were found in the nest surroundings, but no remnants of adults around the predated nests of untagged birds were found. Therefore, our results highlight the importance of scientists considering the potential effects of transmitter devices on data interpretation (Barron et al., 2010).

Compared to the estimates of other studies conducted in farmland, the average number of breeding attempts (1.5) was moderately lower (Daunicht, 1998: 1.8; Jenny, 1990: 1.9–2.3; and Ottens et al., 2013: 2.2). However, we likely missed several breeding attempts that were lost shortly after nest initiation. If Jenny's (1990) calculation that a maximum of 20% of breeding attempts remain undetected also applies to our study area, then Skylarks made on average 1.8 breeding attempts. Comparing our results and previous studies conducted in farmland to estimations of average breeding attempts (2.7) in undisturbed coastal dunes (Delius, 1965), it becomes clear that farmland generally reduces the number of breeding attempts (Donald, 2004). As suggested by Schläpfer (1988), the condition of females in agricultural landscapes likely does not allow a quick restart of breeding after a previous breeding attempt.

Excluding early nest losses, we are confident that we found almost all successful nests of radio‐tagged Skylarks. The regular feeding of chicks is a conspicuous behavior that greatly facilitates the search for nests. Therefore, we believe the documented breeding success, with 0.8 successful nests producing 2.4 chicks that left the nest per pair and year, is reliable for the tagged individuals. The value is similar to the 2.7 chicks per pair and year reported by Schläpfer (1988) and higher than the 1.8 chicks per pair and year documented in Jenny (1990). It is noteworthy that the average breeding success in our study area might have been higher if the radio‐tagging itself reduced nest and female survival. In a Dutch Skylark population with annual return rates of 0.7 for adults and 0.2 for juveniles, three chicks per pair and breeding season are necessary to keep the population stable (Hegemann, 2012; Kuiper et al., 2015). Assuming similar demographic parameters for our study population, the population would be decreasing. However, as the breeding success of tagged individuals strongly varied between 2018 and 2019 for unknown reasons, longer studies are required to make valid predictions about population stability.

Combining our findings on radio‐tagged Skylarks and monitored nests, we contend that the absence of a curtailment of the breeding season results from the advantageous composition of our study area. The two prevailing crops appeared to be suitable nesting habitats for early (winter cereals) and late (sugar beet) breeding attempts. Uncropped land and less frequent summer crops like broad bean also enriched habitat heterogeneity, further reflected in the diverse composition of most Skylark home ranges. The structural diversity of home ranges likely made territorial abandonments or shifts redundant. From our perspective, easy access to safe nesting sites was the prerequisite for consistently high nest success. However, it is also important to note the limitations of our study. The effect of the extraordinarily hot and dry conditions during data collection (Deutscher Wetterdienst, 2021; Zscheischler & Fischer, 2020) could not be analyzed due to the absence of an average reference year. Heat and drought may have directly influenced the breeding success (Reichert et al., 2012) or indirectly by changing the behavior (Buchholz et al., 2019) of interacting species, e.g., an altered pattern of predator activity. Moreover, consideration of intraspecific competition known to affect reproductive output in other bird species (Natsukawa et al., 2019) was beyond the scope of this study. Follow‐up research addressing these issues is thus necessary to further support our interpretations.

## 5 CONCLUSIONS

Our study points to the frequently discussed advantages of heterogeneous farmland for Skylarks and birds in general (Eraud & Boutin, 2002; Flade et al., 2003; Miguet et al., 2013; Püttmanns et al., 2022; Schläpfer, 1988; Tscharntke et al., 2021) by directly analyzing individual fates throughout the breeding season. As Hiron et al. (2012) suspected, winter cereals are not a problem per se but can contribute to a successful breeding season when embedded in a diversified agricultural landscape. Therefore, we believe it is feasible to recommend crop diversification as an effective measure to protect Skylarks in otherwise conventionally managed farmland.

## AUTHOR CONTRIBUTIONS

**Manuel Püttmanns:** Conceptualization (equal); data curation (equal); formal analysis (equal); funding acquisition (equal); investigation (equal); methodology (equal); project administration (lead); visualization (equal); writing – original draft (lead). **Franziska Lehmann:** Data curation (equal); investigation (equal); writing – review and editing (equal). **Fabian Willert:** Investigation (equal); writing – review and editing (equal). **Jasmin Heinz:** Investigation (equal); writing – review and editing (equal). **Antje Kieburg:** Investigation (equal); writing – review and editing (equal). **Tim Filla:** Formal analysis (equal); methodology (equal); writing – review and editing (equal). **Niko Balkenhol:** Conceptualization (equal); supervision (equal); writing – review and editing (equal). **Matthias Waltert:** Conceptualization (equal); supervision (equal); writing – review and editing (equal). **Eckhard Gottschalk:** Conceptualization (equal); funding acquisition (equal); investigation (equal); methodology (equal); supervision (lead); writing – review and editing (equal).

## FUNDING INFORMATION

Deutsche Bundesstiftung Umwelt. Dick Potts Legacy Fund. Fazit‐Stiftung. Naturschutzstiftung Papilio. Stiftung für Ornithologie und Naturschutz. Stöckmann‐Stiftung zur Förderung von Umwelt‐ und Naturschutz.

## CONFLICT OF INTEREST

The authors declare that they have no conflict of interest.

## Data Availability

All datasets and R‐scripts used to generate the results presented in this study are available at the Dryad Digital Repository: https://doi.org/10.5061/dryad.x69p8czmt.
